# Mental Stigmata of Degeneration

**Published:** 1907-08

**Authors:** Arthur Mac Donald

**Affiliations:** Washington, D. C.; Author of Man and Abnormal Man, and Honorary President of the Third International Congress of Criminal Anthropology, of Europe


### Mental Stigmata of Degeneration.

BY ARTHUR MAC DONALD, Washington, D. C.

Author of Man and Abnormal Man,[1] and Honorary President of the Third International Congress of Criminal Anthropology, of Europe.

SOME writers seem to consider all forms of insanity as stigmata of degeneration. But in cases of acute insanity where the cause is due to accidental injury of the head or to toxic effects of disease, or to some moral shock, or excessive mental strain, it would seem questionable to apply to these, or similar cases, the term, "degenerate." Before entering, therefore, into a consideration of the mental stigmata of degeneration, it will be necessary to make reference, as briefly as possible, to classification of different forms of insanity. This is done with a view to finding the place of mental degeneration in such classification and its relation to mentally diseased states in general.

*Classification of Insanity.*—Most alienists group apart the simple forms of insanity known as mania and melancholia. The majority also separate the intermittent forms, as alternate and circulatory insanity and the toxic mental disorders. They isolate also the cerebropathological or organic cerebral affections.

The disagreement commences with the classification of insanity without delirium and delirium proper.

Insanity without delirium includes obsessions, pathological impulsions, and moral insanity, three states which may be considered as stigmata of degeneration.

There are of course difficulties in all classifications of insanity. If we depend upon the symptoms alone, nothing is more variable than the outward changes in the same disease, and nothing is more common than the same symptoms being manifested by different diseases.

Those who classify mental diseases according to their causes are lead to giving undue weight to insignificant and doubtful causes. For the same causes may appear to be present in diseases of different natures and on the other hand, the same disease may seem to be due to several causes.

---

1. Man and Abnormal Man, including a study of children, in connection with bills to establish laboratories under state and federal governments in the study of the criminal, pauper, and defective classes, with bibliographies. Senate Document No. 187, 58th Congress, 3d Session, 780 pages, 8°. Washington, D. C.

The pathological anatomical classification would be ideal, if there were sufficient knowledge of the real causes of different forms of insanity.

Insanity may be divided into two general classes; 1 Hereditary Insanity, that is, where there is a predisposition; and 2, Accidental Insanity, that is, where mental disease is an incident in the life of the normal individual.

But here predisposition can be used only in a relative quantitative sense. For in every disease there must be some slight predisposition or tendency, some soil or spot with little or no resistance.

*Accidental Causes of Insanity.*—An individual can become insane from a series of moral shocks, reverse of fortune, loss of a dear friend, or from an exhausting chronic affection, or acute disease with long and painful convalescence, or from intoxications, as alcoholism or morphinism, and the like. The brain can be put in such a morbid state of weak resistance as to lose its equilibrium at the least unfavorable influence.

*Relation of Insanity to Degeneration.*—But there are numbers of persons who have been subject to such conditions and who have not become insane. There must be a difference between these individuals and the others, and this difference probably is one of predisposition or tendency, no predisposition, or slight tendency to insanity. Those predisposed may be divided into: 1. The predisposed simply. 2. The predisposed with degeneracy.

*The Predisposed to Insanity Simply.*—These have the simple forms of insanity as mania and melancholia, without troubles of the intelligence properly speaking. All these simple forms consist in general delirium, affecting the entire understanding. According to Magnan, there is no intellectuality delirium properly speaking; there are no creations due to aberrations of reason or imagination. There are elementary disorders essentially transitory. Mania and melancholia may be placed with intermediate insanity and chronic delirium.

*Those Predisposed with Degeneracy.*—These have intellectual and moral personality, transformed at its basis through the progressive influence of predisposition.

Those predisposed, but without degeneracy, have a normal brain, but fragile. In these with degeneracy in addition, their mental constitution is at all times abnormal. This fundamental degenerative taint may manifest itself in anomalies of sentiment, intelligence, instincts or inclinations, or it may assume physical anomalies, the signification of which is added to the concomitant mental anomalies. All these stigmata are permanent and born with the individual, and continue until death.

Bad mental, physical, moral or social surroundings can easily develop this degenerative taint. Even physiological moments, such as puberty, menopause, menstruation and pregnancy may make degenerative taint manifest. Here attacks of delirium have no proper evolution, they take all forms and are substituted one for the other, with the greatest facility. Those with the deepest taint are candidates for dementia.

Some of these degenerates may have brilliant minds, but they are without equilibrium; they may be eccentric, bizarre, peculiar and original. They are superior degenerates.

Those with weak minds are distinguished according to degree of weakness as feebleminded, imbeciles and idiots. The transitions between all these divisions are almost imperceptible.

*Classes of Mental Degeneration*[2].—There are three orders or aspects of mental degeneration:

1. States of lucid insanity, with conservation of consciousness at base of obsessions, impulsions or inhibitions.

2. Very advanced states of disequilibrium, with loss of consciousness, generally requiring subject to be confined. To these belong the most dangerous variety as insanity of persecution. Others manifest a deviation or insufficiency of moral sense, and are called morally insane.

3. States of delirium: Delirium, sudden, multiple, polymorphic, protiform rapid or sometimes of long duration, but without tendency to systematisation and progressive transformation. These are curable. All forms of delirium can be observed. To this class belong religious, erotic, hypochondric and ambitious deliriums following the predominant ideas in the individual.

*Remaining Forms of Insanity Without Degeneracy.*—The final forms of insanity following the degenerative forms, are states of delirium directly engendered by neurosis, as hysteria and epilepsy. These states can have a degenerative phase, which pre-exists in the neurosis itself. Sometimes the neurotic delirium co-exists with simple insanity or chronic delirium.

*Divisions of Accidental Insanity.*—Two large divisions of accidental insanity are (a) organic dementia and (b) toxic insanity. These can be referred to their direct causes.

Insanity from intoxications, as alcoholism, absinthism, cocainism, morphinism, etherism, etc., manifest characteristics in common. To their acute forms may be applied the term Hallucinatory delirium.

*Mental Stigmata.*—The characteristic stigma of mental degeneracy is a want of harmony, a lack of balance, a disequilibrium

2. The classification of Magnan is followed in the main.

of the divisions, properties or faculties of the mind, as memory, preception, association of ideas, attention, etc., or generalisation, judgment, and the like.

The faculties of the mental degenerate are out of gear, not intact, their harmony has disappeared. One or more faculties may predominate and some may be so developed as to indicate elements of genius, or others may be absent. A degenerate may be mentally weak, or with exaggerated emotions, or impulsive or obsessed, or have simple tics and spells.

*Evolution of Degeneration.*—The juvenile degenerate learns to talk very slowly, the images of the words do not bcome well fixed in the memory.. The power of synthesis is difficult to educate. There is irregularity of aptitudes and want of equilibrium.

Degenerates are very impressionable, having fears without reason, extraordinary apprehensions in certain things, unreasonable antipathies, excessive sympathies, and nocturnal terrors of every form.

There may be serious morbid motor or sensitive conditions which leave indelible traces, in connection with inflammation of the meninges, resulting in paralysis, spasms, convulsions, hemiplegia, hemichorea, symptomatic epilepsy, and the like.

From infancy to puberty, the degenerate remains somewhat the same. His mental weakness may reveal itself at school. Or if his intelligence is acute, it is unequally developed, making the child a little prodigy in some ways, but defective in other respects.

*Puberty.*—In puberty sexuality is predominant. At menstruation there is moodishness, nervousness, or hysteria, obsessions or menstrual insanity. In both sexes, there is frequent onanism, a cause of exhaustion. The sexual function may be previous or sometimes neutral if not absent.

*Adolescence.*—In adolescence the degenerate is thrown more upon his own resources, he shows himself more than ever insufficient for life's struggle, for which he is poorly fortified and which produces in him pathological results. With a weak intelligence and lawless imagination, he may show the most foolish enthusiasm, burlesque inventions, religious exaggerations to mysticism or to asceticism. Credulous and superstitious, he submits to all suggestions and becomes a victim of contagion. Laws, morality, the most respected customs, he revolts against as a restriction of his liberty; not comprehending their purpose. He is an easy victim to alcohol, morphine, cocaine, and the like.

*Degenerate Easily Exhausted.*—Life's constant struggle soon exhausts the degenerate. Extreme mental fatigue results, all labor is impossible, where accompanied with pain: he has neurasthenia, a state of irritable weakness. In the most serious cases, the degenerates become insane; mental equilibrium is broken, reason totters, conscience is obscured, chaos reigns in thoughts, sentiments and acts, revealing what may be called the maximum of disequilibrium.

*Idiocy.*—The lowest type of degenerate with the most rudimentary intelligence is the idiot. His sensitive centres are scarcely developed, his sensations are narrow, and perceptions almost nil. Mentally, there is an arrest of development, the mind can do nothing of itself; life is a succession of reflexes, which do not relate to the psychical centres. The idiot is an instinctive being. He defends himself like the most inferior animals, he never acquires personality, he is an automaton. The idiot of the lowest degree is apathetic and unable to express his desires by speech or motion. The idiot of a little higher grade may be excitable, mischievous, violent in temper, restless, willful and very curious. Speech is delayed or limited to simple words, incomplete sentences or short phrases.

*Inferior Feeblemindedness.*—The next rank above the idiots is occupied by the inferior feebleminded. These have more intelligence than the idiot but it is very variable and incomplete. From the idiot up to the intelligent degenerate, are a number of stages. The most inferior are very near the idiot but very different from the most superior who in turn are very near the intelligent degenerate. In the inferior feebleminded, the elementary intellectual faculties can reach their full development. This class are still imbeciles, they have only a minimum intelligence, approaching that of the animals.

*Superior Feeblemindedness.*—The superior feebleminded person can be a brilliant individual, offering all the elusions of a weighty intelligence, but of which the insufficiency will never fail to show itself wherever the judgment formulates the conclusions. He can collect isolated facts but cannot apply or classify them. He is not greatly different from the normal child. He may be precocious, with a great capacity for remembering dates, or for arithmetical calculations, the wonder of the family.

*Intelligent Degenerates.*—In the intelligent degenerates, the higher faculties acquired much development, but very unequal, as the variety of bisarre adaptations of these faculties shows. This class possess the necessary mental qualities to become of importance, but they are unable to direct their thought and acts continually to an end. They cannot coordinate the powers they have. They may reason correctly, but their actions are most incoherent.

Sometimes without motive, fluctuating and undecided, they manifest obstinancy that nothing can influence. Their disequilibrium may result from excessive development or from the richness of certain faculties. There may be strong imagination coexisting with narrow conceptions; or high morality with sincerity, and at the same time most indelicate acts. The intelligent degenerate may not be able to control and regulate his sentiments, affections and impulses. In spite of his intellectual development he can be the toy of his lowest passions and instincts. Thus the intelligent degenerate can be rightly called, bizarre, hair-brained, eccentric and original. It is clear, that such an individual without equilibrium, yet with an active mind is liable to come in conflict with established customs, if not with the law itself.

*Obsessions and Impulsions.*—Obsession and impulsion are perhaps the highest and most significant expression of degeneracy. They have been made morbid entities under different names as emotional delirium, insanity of doubt, mania of theft, mania of suicide, etc.

Every idea imposing itself upon consciousness, in spite of the will, thus interrupting the regular course of thoughts, is an obsession.

Every act consciously performed, but which cannot be inhibited by the will, is an impulsion.

The obsessed degenerate has no emotional equilibrium, sensations, images and perceptions impress themselves with such intensity, as to produce inhibition, characteristic of conscious obsession. The disorders of sentiment are the most complex; they may result in exaggerated altruism, or a foolish and injurious philanthropy. Or there may be an excessive egoism; or desire to satisfy some passion, dominating all other sentiments.

*Pathological Obsession.*—A pathological obsession is a morbid syndrome characterised by the sudden appearance of an idea, imposing itself upon consciousness in the form of paroxysms, interrupting the normal state of the mind in spite of the will, whose powerlessness is turned into intense agony and moral suffering.

As to the different forms of pathological obsession and impulsion it may be said that they are summarised in insanity of doubt, echolalia aboulia, and coprolalia.

*Insanity of Doubt.*—This is an obsession in the form of mental questioning. Consciousness is clear. The questions arise in the mind in spite of the will, which is agonising. When the question has received an answer, there is a momentary relief. The questions are of every kind and some are most insignificant.

In his pathological doubt, the person is constantly preoccupied with the solution of these problems; he cannot do anything else, his doubts cause him agony which renews itself in a paroxysm from the appearance of a new series of questions and doubts.

The person is perfectly conscious of his situation; he makes desperate efforts to turn his attention away from objects he declares to be foolish; but all is in vain. Every minute brings new doubts and new efforts; the person has much moral suffering at the moment of the paroxysms. This suffering is sometimes relieved by some answer, but it is only temporary, the obsession returns with more intensity than ever. This suffering has its physical signs, the forehead is covered with perspiration, the pulse increased; there are palpitations, precardial pains, a pain in the frontal region, indicating excess of attention and intellectual fatigue.

One of the forms of insanity of doubt is kleptophobia, or the fear of stealing, which may assume all the morbid characteristics of insanity of doubt.

*Aboulia.*—In aboulia, the degenerate feels his will-power suddenly destroyed, at the very instant it is necessary for him to carry out some determination. When ready to sign a paper to end an account, start out to walk, or in any condition important or otherwise, the degenerate finds himself suddenly hindered, the act willed is not performed, or if commenced, it is not achieved. All this time the mind is perfectly clear. No plausible reason exists apparently to explain the phenomenon. An irresistible hand seems to hold the degenerate back, so long as he struggles.

*Echolalia.*—Echolalia is a simple impulsion to repeat in spite of oneself, words, phrases, or last words or phrases, which one hears. There is nothing intellectual here, it is a mere echo. The image of the word awakens immediately the motor image of articulation and even at the moment the sensorium is warned, or before it can be appreciated, this image is objectified. The person not being able to prevent this, is seised with great anxiety. He is especially anxious at the thought of the necessary return of the impulsion. He flees the world, avoids all conversation and becomes mute.

*Coprolalia.*—Certain degenerates are sometimes obsessed with obscene words, which arise suddenly in consciousness, without being caused by any association of ideas. This is called coprolalia.

These ideas have no relation to anything, and yet thrust themselves upon the attention, interrupting the regular course of thought. It is a stranger who comes to trouble the mind. Anxiety is at its height, the degenerate knows the effect which the obscene words will produce and at the same time is conscious of his powerlessness to prevent what he feels is coming. The paroxysm approaches, perspiration is upon his brow, his face is full of agitation, his heart beats violently. Suddenly the word is forced out.

Then there is a feeling of relief and calm. No word can express the moral torture these degenerates suffer.

*Delirium of Touch.*—The subject is obsessed with fear of contact with certain objects; it is impossible to overcome this fear or repugnance. The effort, or struggle to do so, is agonising. A momentary victory gives satisfaction.

A form of this delirium is pyrophobia, or fear of fire, or the fear of objects that can be set on fire. The fear of setting fire to, is a form of the insanity of doubt and is accompanied with cruel agony; the idea cannot be chased away without a long struggle. The mind is clear.

*Terrors of Space.*—There are three forms of terror or fear (a) an obsessed and insurmountable fear of spaces called *agoraphobia,* (b) a terror of restricted or limited spaces, called *claustrophobia* and (c) a fear of known places or determined locations, designated *topophobia,* all of which are stigmata of degeneration.

*Dipsomania and Sitiomania.*—In dipsomania the impulsion to drink is irresistible. The struggle may be energetic, but it is useless against the temptation. The mind is clear, the suffering is extreme. There is relief when the impulsion has been satisfied.

Sitiomania is an impulsion similar to dipsomania with like symptoms in respect to food.

*Pryomania and Kleptomania.*—In both pyromania and kleptomania the impulsion is irresistible, the mind is clear; the struggle is painful, in one, to resist setting on fire, in the other, to resist stealing. In both there are physical symptoms, and satisfaction on the accomplishment of the acts.

*Impulsions to Homicide and Suicide.*—In homicide,. the temptation to kill an innocent or indifferent person, or even a dear friend, without motive, is irresistible. The moral suffering of the degenerate is indescribable, his continued struggle terrible. The physical reaction is marked. If the act be accomplished it is followed by a feeling of relief, even though the sentiment of horror remain in consciousness.

The suicidal impulsion presents the same symptoms as that of homicide.

*Arithmania and Onomatomania.*—In arithmania there is an irresistible impulsion to count, which at times is so intense that the person counts automatically. In the paroxysms in the midst of the conscious effort to struggle against the impulsion, there arises intense suffering with habitual physical symptoms. Numeration alone can give relief.

Onomatomania has numerous forms. The most common is an obsessed and agonising search for certain words. The mind is clear. The degenerate endeavors to chase away the idea, which he recognises as absurd; but the obsession repeats itself, being accompanied with extreme anxiety and physical signs. The finding of the word brings the desired relief.

*Oniomania and Mania for Play.*—The impulsion to buy all sorts of things is oniomania. This impulsion is painful, but invincible, in spite of all effort, the accomplishment of which brings relief.

Mania for play, sport, or game is a condition, where the player is pushed in spite of his resistance to play. Conscious of his condition, which he deplores, he struggles and suffers with certain defeat before him. The accomplishment of the act is accompanied with violent emotions followed by satisfaction, mingled with regrets.

*Sexual Perversions, Aberrations and Anomalies.*—The number of sexual morbid states included here is enormous.

With the exception of cases where the sexual perversion is moral and so ignored by the degenerate himself, all other cases show the general characteristics of obsession and impulsion.

